# Ciliary Entry of the Hedgehog Transcriptional Activator Gli2 Is Mediated by the Nuclear Import Machinery but Differs from Nuclear Transport in Being Imp-α/β1-Independent

**DOI:** 10.1371/journal.pone.0162033

**Published:** 2016-08-31

**Authors:** Belén Torrado, Martín Graña, José L. Badano, Florencia Irigoín

**Affiliations:** 1 Human Molecular Genetics Laboratory, Institut Pasteur de Montevideo, Mataojo 2020, Montevideo CP11400, Uruguay; 2 Bioinformatics Unit, Institut Pasteur de Montevideo, Mataojo 2020, Montevideo CP11400, Uruguay; 3 Departamento de Histología y Embriología, Facultad de Medicina, Universidad de la República, Gral. Flores 2125, Montevideo CP11800, Uruguay; University of Toronto, CANADA

## Abstract

Gli2 is the primary transcriptional activator of Hedgehog signalling in mammals. Upon stimulation of the pathway, Gli2 moves into the cilium before reaching the nucleus. However, the mechanisms underlying its entry into the cilium are not completely understood. Since several similarities have been reported between nuclear and ciliary import, we investigated if the nuclear import machinery participates in Gli2 ciliary entry. Here we show that while two conserved classical nuclear localization signals mediate Gli2 nuclear localization via importin (Imp)-α/β1, these sequences are not required for Gli2 ciliary import. However, blocking Imp-mediated transport through overexpression of GTP-locked Ran reduced the percentage of Gli2 positive cilia, an effect that was not explained by increased CRM1-dependent export of Gli2 from the cilium. We explored the participation of Imp-β2 in Gli2 ciliary traffic and observed that this transporter is involved in moving Gli2 into the cilium, as has been described for other ciliary proteins. In addition, our data indicate that Imp-β2 might also collaborate in Gli2 nuclear entry. How does Imp-β2 determine the final destination of a protein that can localize to two distinct subcellular compartments remains an open question. Therefore, our data shows that the nuclear-cytoplasmic shuttling machinery plays a critical role mediating the subcellular distribution of Gli2 and the activation of the pathway, but distinct importins likely play a differential role mediating its ciliary and nuclear translocation.

## Introduction

The primary cilium is a cellular organelle whose importance in cell physiology has been evidenced only recently, based on observations linking its dysfunction with several human pathologies grouped under the name of ciliopathies [1,2]. The primary cilium protrudes from the cell surface and acts as an antenna for sensing and transducing a myriad of extracellular signals [3,4]. Although the intraciliary compartment and the ciliary membrane are continuous with the cytosol and plasma membrane respectively, the cilium is composed by a characteristic set of proteins [5], indicating the existence of specific mechanisms for protein targeting and transport to the cilium. At the base of the cilium, a complex network of membrane and soluble proteins, the transition zone (TZ), constitutes a selective gate for ciliary proteins. Highlighting the importance of this structure in the control of protein transport and cilia function, mutations in a number of TZ proteins have been shown to cause severe ciliopathies, such as Meckel-Gruber and Joubert syndromes and nephronophthisis [6,7]. However, little is known about specific mechanisms of protein targeting and movement across the TZ, especially in the case of soluble ciliary proteins.

Recent reports have shown striking similarities between the movement of proteins from the cytosol to the nucleus and to the cilium, to the extent of comparing the TZ with a “ciliary pore” analogous to the nuclear pore complex [8,9]. Conventional nuclear transport of proteins larger than 45 kDa is mediated by karyopherins, a protein family functionally divided into importins (Imps), which move proteins into the nucleus, and exportins that move proteins in the opposite direction. In the cytoplasm, Imps bind cargo proteins harbouring nuclear localization signals (NLS) and the assembled complex crosses the nuclear pore complex by the interaction of Imps with nucleoporins. Once inside the nucleus, the Imp interacts with the small G-protein Ran bound to GTP and releases the cargo. Then, Imp-RanGTP moves back to the cytoplasm where GTP is hydrolysed, disassembling the complex and releasing the Imp for another round of transport. Exportins work in an analogous way albeit in the reverse direction, as cargo binding occurs in the nuclear compartment and depends on the complex exportin-RanGTP. Thus, the directionality of transport relies on the existence of a Ran gradient, whereby RanGTP is enriched in the nucleus while RanGDP prevails in the cytoplasm. This gradient is formed and maintained through the action of the chromatin-associated RanGDP-GTP exchange factor (RCC1) and cytoplasmic RanGTPase-activating proteins (RanBP1 and RanGAP) [10]. There are at least 10 different Imps in mammals [11], Imp-β1 being the best characterized of them. Imp-β1 associates with Imp-α, which recognise "classical NLS" (cNLS) consisting of one (monopartite) or two (bipartite) stretches of basic amino acids [12]. Such motifs can be found by specific algorithms. In contrast, the NLSs recognized by Imp-β2 (PY-NLS) are more diverse and their prediction from protein primary sequence alone remains elusive [12].

The movement of proteins to the cilium resembles nuclear transport in several aspects: i) there is restricted diffusion into the ciliary compartment of proteins larger than a certain threshold (60–100 kDa) [8,13], ii) nucleoporins are present at the ciliary base [8,14], iii) Imp-β2 has been shown to be used for the movement of certain ciliary proteins [15,16] and in these cases directionality of transport is governed by the unequal distribution of RanGTP/RanGDP, with RanGTP being enriched within the cilium [15]. Interestingly, several proteins have been localized in both the nucleus and cilium [17–20], but it is not known whether the same machinery participates in their transport to both compartments. If this was the case, additional mechanisms should exist to determine the final destination of these proteins.

One example of proteins presenting this nuclear/ciliary localization pattern is the Gli transcription factors of the Hedgehog (Hh) pathway. The Hh pathway in vertebrates has the peculiarity of using the primary cilium as a signalling platform, compartmentalizing the transduction process and providing extra possibilities for regulation and fine-tuning [21]. Hh signalling involves different players that change their subcellular localization, entering and/or leaving the cilium, in a tightly regulated but still not completely understood manner [18,22–25]. Here we focussed on GLI-family zinc finger-2 (Gli2), the major transcriptional activator of Hh signalling in mammals. Gli2 activity is regulated by several mechanisms that have been extensively studied (for in depth recent reviews on the topic see for example references [24,26]). Briefly, in the absence of Hh ligand, Gli2 is inhibited mainly in two ways: i) PKA-driven Gli2 phosphorylation induces Gli2 ubiquitination and degradation through the proteasome [27,28] and ii) Gli2 is bound to SuFu, a negative regulator of the pathway that sequesters Gli2 in the cytoplasm [29–31]. Upon pathway activation the degradation of Gli2 is inhibited [27] and the protein accumulates at the ciliary tip before translocating into the nucleus to control the expression of target genes [23,31]. Within the cilium Gli2 dissociates from SuFu and becomes transcriptionally active [30,31]. Both Gli2 accumulation within the cilium and its activation are strictly dependent on ciliary, active Smoothened (Smo) [23,31]. However, the mechanism that targets and mediates Gli2 entry into the cilium is still not known. In fact, this issue remains unsolved for many other soluble proteins destined to the cilium [6].

Here we show that although the nuclear-cytoplasmic shuttling machinery is used both for nuclear and ciliary localization of Gli2, the mechanisms for these two transport events differ. Whereas nuclear import requires two cNLSs in Gli2 and the complex Imp-α/β1, ciliary localization requires neither of these, and instead uses Imp-β2.

## Materials and Methods

### DNA constructs and generation of Gli2-NLS mutants

pEGFP-C (Clontech) coding for *Mus musculus (Mm)_*Gli2 was kindly provided by Dr. Amin Liu (Department of Biology, Eberly College of Science, The Pennsylvania State University). Plasmids coding for Cerulean or myc-tagged RanWT or RanG19V were a gift from Dr. Kristen J. Verhey (Department of Cell and Developmental Biology, University of Michigan Medical School). Plasmids coding for myc-MBP or myc-MBP-M9M were kindly supplied by Dr. Yuh Min Chook (Department of Pharmacology, University of Texas Southwestern Medical Center, Dallas). To generate the Gli2 NLS mutants, mutNLS-1 (R225A, K226A), mutNLS-2 (K557A, K578A) and the double mutant mutNLS-1+2 (R225A, K226A, K557A, K578A), we designed primers introducing the specified changes and performed site-directed mutagenesis using the Quick Change Site-Directed Mutagenesis Kit following the manufacturer’s instructions (Stratagene). Primers are available upon request.

### Cell culture and treatments

NIH/3T3 (ATCC) cells were cultured in Dulbecco’s modified Eagle medium (Life) supplemented with 10% calf bovine serum (CBF, Life), penicillin (100 U/ml) and streptomycin (100 μg/ml) at 37°C in 5% CO_2_. Stable cell lines obtained as described below were maintained in the same medium but in the presence of the selection agent. Cell transfections with plasmids were performed using Turbofect (Fermentas) at a ratio of 1ug DNA:2 μl Turbofect as suggested by the manufacturer. Activation of Hh signalling was performed on cells previously cultured in 0.5% CBS for 24 h in order to promote ciliation by incubation with: a) 400 nM SAG (Enzo), a cell-permeable clorobenzothiophene that modulates the coupling of Smo with its downstream effectors acting as an agonist of the pathway [32], for 90 min, or b) conditioned medium containing an active N-terminal fragment of the Shh molecule (Shh-N) obtained as described previously [33], diluted 1:2 with fresh medium lacking CBS, for 3 or 18 h depending on the experiment. Control cells were incubated with equal volumes of DMSO or control medium respectively. The pRK5 plasmid coding for Shh-N was kindly provided by Dr. Philip Beachy (Institute for Stem Cell Biology and Regenerative Medicine, Stanford University). In the experiments using importazole (IPZ, Sigma), cells were incubated with the drug at 40 μM 1 h before addition of SAG and control cells were treated with DMSO. In the experiments using MG132 (Sigma) cells were incubated with the drug at 50 μM for 90 min. In the experiments using leptomycin B (LMB, Sigma), cells were incubated with the drug at 40 nM in methanol, at the same time of Hh stimulation with SAG or the control condition.

### GFP-Gli2 stable cell lines

NIH/3T3 stable cell lines expressing GFP-*Mm*_Gli2 or the NLS-mutants were obtained by lentivirus transduction. Coding sequences of GFP-Gli2 and GFP-mutNLS Gli2 were amplified from pEGFP-C vectors and cloned into pLJM1 (a gift from Dr. David Sabatini, Whitehead Institute for Biomedical Research, Cambridge, MA) using SalI and MluI. Lentiviruses were obtained by transfecting HEK293FT cells with pLJM1-GFP-Gli2 and the plasmids pCMV-dr8.2 and pCMV-VSVG as described previously [34]. Virus-containing supernatants were collected 72 h post-transfection, cleared by centrifugation, filtered through 0,45 μm and stored at -80°C until used. NIH/3T3 were infected with lentiviruses (MOI of 5–10) in media containing 8 μg/ml polybrene (Sigma). Twenty four hours later infected cells were selected with 2 μg/ml puromycin (Life) and analysed by assessing SAG-induced Gli2 ciliary localization. The other stable cell lines expressing GFP-*Mm*_Gli2 or GFP-*Mm*_Gli2-mutNLS-1+2 were generated using the Flp-In method according to the manufacturer’s recommendations (Life). Briefly, NIH/3T3-Flp-In (Life) were transfected with pOG44 and the pEF5/FRT/V5-D-TOPO vector where we cloned the GFP- *Mm_*Gli2 or mutNLS-1+2 coding sequences. After 2 days, cells were seeded at low density and culture medium was supplemented with hygromycin (150 μg/ml) for stable integrant selection. Cells were analysed by comparing the levels of GFP-Gli2 with those of endogenous Gli2 by western blot and the response to SAG stimulation was evaluated by analyzing GFP-Gli2 ciliary localization.

### Immunoprecipitations

Cells were lysed in buffer containing 10 mM Tris/HCl, pH 7.5, 150 mM NaCl, 0.5 mM EDTA, 0.1% Triton X-100 supplemented with 1x protease inhibitor cocktail (Sigma), phosphatase inhibitors (0.5 mM Na_3_VO_4_ and 50 mM NaF) and 25 μM MG132. Cell lysates (2 mg of protein at a final concentration of 1 mg/ml) were pre-cleared using uncoupled agarose beads (ChromoTek) and then incubated with GFP-Trap beads (ChromoTek), 25 μl of a 50% slurry. Binding was performed at 4°C for 2 h under rotation. Beads were extensively washed with ice-cold lysis buffer and washing buffer (the same as lysis buffer but without Triton X-100) and finally resuspended in 6x SDS sample buffer and analyzed by SDS–PAGE and western blotting. Cell lysates were obtained from HEK293FT cells transfected with 11 μg pEGFP-C empty vector or pEGFP-Gli2 per 3.3x10^6^ cells, or from NIH/3T3 Flp-In stable cell lines expressing GFP-Gli2 wt or mutNLS-1+2. In this latter case cells were grown in 150 mm dishes, cultured 24 h with 0.5% CBS previous activation of Hh signaling with 400 nM SAG for 3 h. As a control for unspecific binding lysates were obtained from NIH/3T3 Flp-In cells transfected with pEGFP-C empty vector.

### Western blot

Cells were lysed in buffer containing 50 mM Tris/HCl, pH 7.4, 150 mM NaCl, 1% NP-40, 0.5% sodium deoxycholate, 5 mM EDTA, and 1 x protease inhibitors (Sigma). The lysates were cleared by centrifugation at 17,000 *g* for 20 min and protein concentration was determined using the BCA assay (Thermo). 60–100 μg of total protein was separated by SDS-PAGE on 8% gels and transferred to PVDF membranes for western blotting. The primary antibodies used were anti-Gli2 (H-300 rabbit polyclonal, Santa Cruz Biotechnology, 1:500, or goat polyclonal, R&D Systems (AF3635), 1:1000, overnight at 4°C), anti-GFP (rabbit polyclonal, Life or Cell Signaling Technology, or rat polyclonal, ChromoTek, 1:1000, 2 h at room temperature), anti-Imp-α1(#MAB6207) and Imp-β1 (#MAB8209) (mouse monoclonals, R&D Systems, 1:7000 and 1:1000 respectively, overnight at 4°C), anti-Imp-β2 (mouse monoclonal, Abcam #ab10303, 1:1000, overnight at 4°C) and anti-α tubulin (mouse monoclonal, Sigma, 1:2000, 1 h at room temperature). Secondary antibodies were anti-rabbit IgG-HRP (goat polyclonal, Sigma, 1:50,000), anti-mouse IgG-HRP (goat polyclonal, Santa Cruz Biotechnology, 1:10,000) and anti-rat IgG-HRP (goat polyclonal, Sigma, 1:4000), and were incubated 1 h at room temperature. Detection was performed using the Pierce ECL Western Blotting Substrate (Thermo). The quantification of western blot bands was done using the open-source software Fiji (National Institute of Health).

### Evaluation of the effect of IPZ and M9M on NFAT and hnRNPA1 nuclear import

The transcription factor NFAT and the heterogeneous nuclear ribonucleoprotein A1 (hnRNPA1) were used as positive controls for Imp-β1 and Imp-β2 mediated nuclear import respectively [35,36]. The effect of IPZ in inhibiting Imp-β1 was first tested on NIH/3T3 transiently transfected with pEGFP-C-NFAT (kindly provided by Dr. Rebecca Heald, Molecular and Cellular Biology Department, University of California, Berkeley). Eighteen hours post transfection NFAT nuclear translocation was stimulated by rising intracellular calcium with 1.25 μM ionomycin (Life) for 15 min. Cells were then processed for immunofluorescence as described below. When IPZ was used, cells were incubated with 40 μM of the drug 1 h before adding ionomycin.

In the experiments analyzing the effect of M9M peptide, cells were co-transfected with plasmids coding for myc-MBP or myc-MBP-M9M together with either pEGFP-C-NFAT or pcDNA3.1-Flag-hnRNPA1 (kindly provided by Dr. J. Paul Taylor, Department of Developmental Neurobiology, St Jude Children’s Research Hospital, Memphis). Eighteen hours post transfection NFAT nuclear translocation was stimulated as previously described and cells were prepared for immunofluorescence.

### Immunofluorescence, confocal microscopy and image analysis

Gli2, NFAT and hnRNPA1 subcellular localization as well as identification of Ran or MBP/MBP-M9M expressing cells were determined by immunofluorescence. Briefly, cells were fixed with 4% PFA for 10 min at room temperature, permeabilised with 0.1% TritonX-100 for 10 min and blocked with 5% FBS. For Gli2 localization in cell lines expressing GFP-Gli2 we used a rabbit polyclonal anti-GFP (Life, 1:1000, 1 h at room temperature) and in normal NIH/3T3 cells expressing Ran-Cerulean, we localized the endogenous Gli2 using a rabbit polyclonal anti-Gli2 (H300, Santa Cruz Biotechnology, 1:100, overnight at 4°C). GFP-NFAT was visualized using the GFP fluorescence and hnRNPA1-Flag was detected using a mouse monoclonal anti-Flag (Sigma, 1:1000, 1 h at room temperature). Primary cilia were stained using a mouse monoclonal anti-acetylated tubulin (Ac.Tub) (Sigma, 1:1000, 1 h at room temperature) and a goat polyclonal anti-myc (Abcam, 1:1200,1 h at room temperature) was used for detection of myc-Ran (WT or G19V), myc-MBP and myc-MBP-M9M. Nuclei were stained for 1 h at room temperature with TOPRO (Life, 1:500) or DAPI (Life, 1:5000). Secondary antibodies (Life) were incubated at 1:1000 for 1 h at room temperature: anti-rabbit IgG-AF488 (donkey polyclonal), anti-mouse IgG-TMRM (goat polyclonal), anti-mouse IgG-AF647 (donkey/goat polyclonal), and anti-goat IgG-AF594 (donkey polyclonal).

Images were obtained in a Leica TCS-SP5 confocal microscope using a 63x oil 1.4 NA objective, Z-sections of 0.5 μm of thickness, and acquired with identical gain, offset and laser power settings. Images were processed and analysed using Fiji. Images shown in the figures are representative of the results obtained in different experiments. For analysing protein localization (Gli2, NFAT or hnRNPA1) we made composite images and obtained the Z-projection (sum algorithm). The fraction of Gli2 positive (Gli2+) cilia was determined counting the number of cilia with Gli2 staining at the ciliary tip over the total number of ciliated cells. In experiments that included transfected cells the analysis was done over the ones that were both, transfected and ciliated. Results were expressed as the fraction of Gli2+ cilia with 95% confidence interval (95% CI), calculated as $\hat{p}\pm z_{a∕2}\sqrt{\frac{\hat{p}\left(1-\hat{p}\right)}{n}}$ [37].

The amount of the protein of interest in a particular subcellular compartment was estimated measuring on a Z-projection (sum algorithm) of the channel where the protein was visualized, the mean fluorescence intensity of manually defined regions of interest (ROIs). In the case of the nucleus, the ROI was defined on the channel corresponding to DAPI or TOPRO, and in the case of Gli2 at the ciliary tip the ROI was defined using the channels of acetylated tubulin and Gli2. The mean fluorescence intensity from the nucleus and ciliary tip was normalised against the mean fluorescence intensity of the whole cell, so as to correct for variations in Gli2 expression among different cells. Results are expressed as box plots where the 25–75% quartiles are drawn using a box, the median is shown with a horizontal line inside the box and the minimal and maximal values are shown with short horizontal lines. Cilia length measurements were also done defining ROIs in the acetylated tubulin channel with freehand lines. The fraction of ciliated cells was calculated counting the number of cilia over the total number of cells. Results were expressed as the fraction of ciliated cells with 95% CI.

### Hh reporter Luciferase Assay

The Hh reporter luciferase assay was performed as described previously [38]. Briefly, NIH/3T3-Flp-In cells were transfected with a total of 1.2 μg DNA and 2.4 μl Turbofect. DNA comprised 50% construct of interest (300 ng pEGFP-Gli2 and/or 100 ng myc-MBP-M9M) or controls (300 ng pEGFP-empty vector and/or 100 ng myc-MBP), 47.5% of Hh reporter plasmid (8x3' Gli-BS-delta51-LucII [39] provided by RIKEN BRC, which is participating in the National Bio-Resources Project of the MEXT, Japan) and 2.5% of pIS1 as the transfection control. The plasmid pIS1 was a gift from David Bartel (Addgene plasmid # 12179) and was derived from pRL-TK by adding more cloning sites. After reaching confluence the cells were serum-starved and treated for 30 h with vehicle or 400 nM SAG. IPZ was added 2 h and 30 min before the end of the assay. The luciferase assay was performed using the Dual Luciferase Reporter Assay System (Promega) according to the manufacturer’s instructions. All luminescence values were background-corrected using readouts from untransfected cell lysates and for each sample the relative luminescence units (RLU) were calculated as the ratio of the firefly luciferase luminescence value (Hh-dependent) divided by the renilla luciferase luminescence value (Hh-independent). Results are expressed as RLU of samples stimulated with SAG or overexpressing Gli2/RLU of control samples. All plots are mean ± SD of triplicates and the assay was repeated twice.

### Statistical Analyses

The fraction of Gli2+ cilia or ciliated cells was expressed as a proportion with 95% confidence interval. In these cases, comparison between different treatments was done using a test of hypothesis specific for comparison of two proportions (hypothesis test for proportions) as described [37]. In the case of measurements of mean fluorescence intensities, or cilia length, sets of data were first tested for normal distribution, using the Shapiro-Wilk test, and variance homogeneity using the Levene test. When necessary, as in the case of Gli2 nuclear fluorescence, data were Ln transformed in order to meet distributional assumptions required by the statistical tests. To compare three or more groups with normal distribution we used one-way ANOVA with Tukey’s post hoc test. In the case of data that do not follow normal distribution we used non parametric tests, the two-tailed Mann Whitney in order to compare two sets of data or the Kruskal Wallis when we had to compare more than two sets. Significance was assumed when p < 0.05. In multiple comparisons Bonferroni correction was applied, the cut-off for statistical significance was reduced from 0.05 to a value determined by the number of comparisons [37].

## Results

### Conserved cNLSs and Imp-α/β1 mediate Gli2 nuclear import

**Fig 1:**
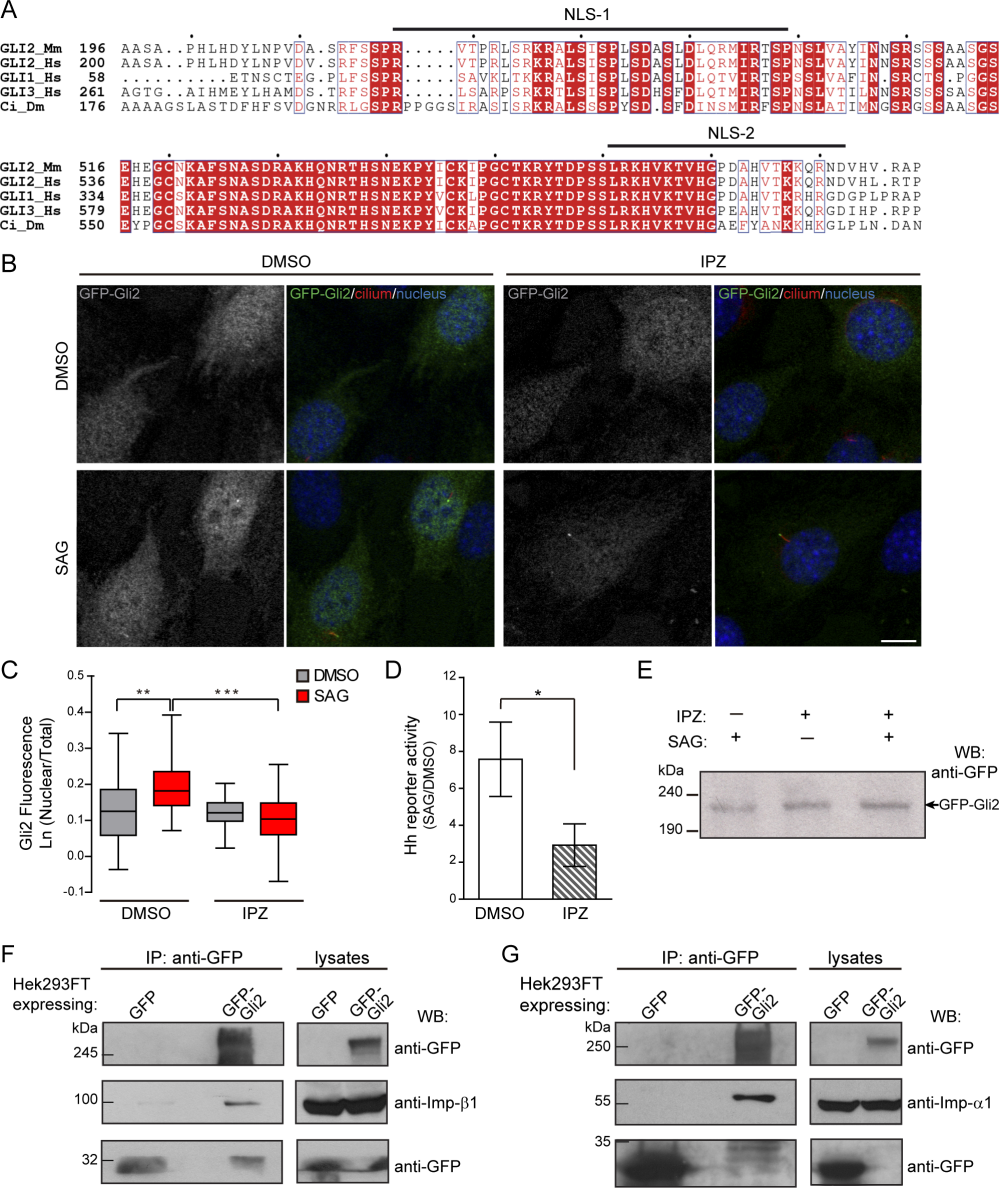
Imp-α/β1 transports Gli2 into the nucleus. (A) Two segments of a T-Coffee [69] alignment of four Gli-like sequences: GLI2_*Mm* (*Mus musculus*), GLI2_*H*s, GLI1_*Hs*, GLI3_*Hs* (*Homo sapiens*) and Ci_*Dm* (*Drosophila melanogaster*). Residue similarity is colour coded according to the Risler substitution matrix, using ESPript [70]. Dots mark 10-residue intervals of the top sequence. Black bars indicate the two bipartite cNLS (cNLS-1 and cNLS-2) predicted by cNLS mapper [40] for GLI2_*Mm*. (B) GFP-Gli2-NIH/3T3 cells were treated with Importazol (IPZ) in order to inhibit Imp-β1-mediated nuclear transport. DMSO was used as control. Hh signalling was activated for 90 min using SAG and non-stimulated cells were treated with DMSO. Cells were stained for cilium (anti-Ac.Tub, red), GFP-Gli2 (anti-GFP, green) and nucleus (TOPRO, blue). The same pictures are also shown with the channel corresponding to GFP-Gli2 in grey scale to help the visualization. Scale bar: 10 μm. (C) Quantification of nuclear Gli2 was performed as explained in Materials and Methods. The mean nuclear fluorescence was normalised against the mean total GFP fluorescence of the cell, so as to correct for variations in Gli2 expression among different cells. Results are representative of three experiments and at least 60 cells were analysed for each condition. ** p<0.001, *** p<0.0001 (Kruskal-Wallis test). (D) Activation of a luciferase-based Hh reporter gene in NIH/3T3 cells stimulated with SAG (or DMSO as control) in the presence of IPZ or DMSO. As explained in Materials and Methods, RLU values from SAG treated cells are normalised against RLU of DMSO treated ones and expressed as mean ± s.d. from triplicates from two independent experiments. * p< 0.01 (Mann-Whitney test). (E) Western blot (WB) showing GFP-Gli2 levels in control and IPZ-treated cells. GFP-Gli2 was detected using an anti-GFP antibody. (F-G) WB detecting Imp-β1 (F) and Imp-α1 (G) after precipitating GFP-Gli2 with GFP-Trap (left) from HEK293FT transfected with pEGFP-Gli2 (left panels). Membranes were cut at different levels so as to detect in the same samples the precipitated GFP-Gli2 and GFP (left panels). The levels of GFP-Gli2, Imp-β1, Imp-α and GFP in the lysates used for immunoprecipitation were assessed by WB and are shown in the right panels. The band corresponding to GFP looks distorted because the protein migrates with the dye front.

**Fig 2:**
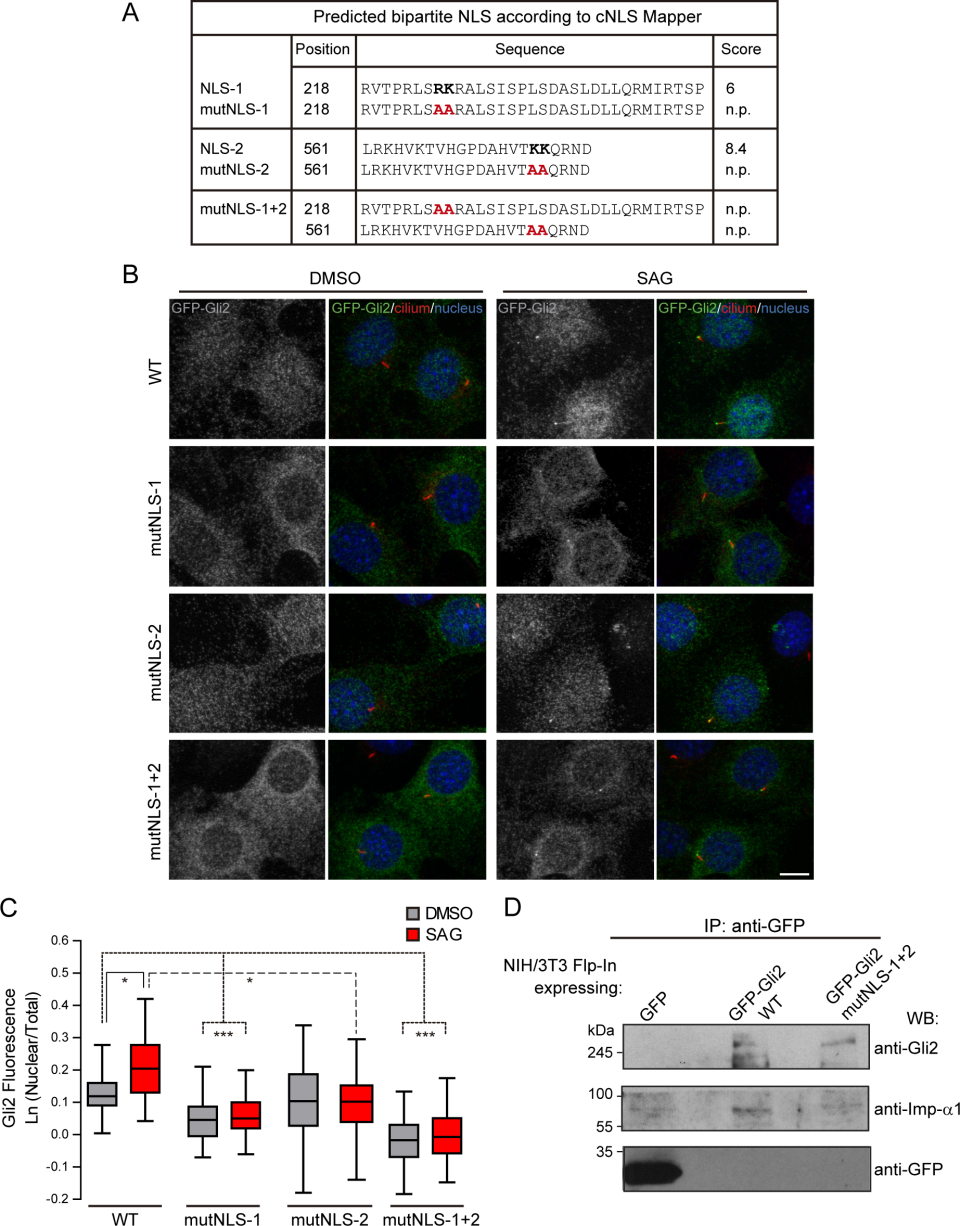
cNLSs are necessary for Gli2 nuclear import. (A) Sequences in Gli2_*Mm* predicted as cNLSs by cNLS-Mapper [40] using a cut-off of 5 with the corresponding scores (out of 10). Residues that were mutated for alanines in the mutNLS constructs are in bold. The mutated sequences are not predicted (n.p.) as cNLS. (B) Transduced NIH/3T3 cells expressing GFP-Gli2, wt or cNLS mutants (mutNLS-1, mutNLS-2 or mutNLS-1+2), were treated with SAG for 90 min or DMSO as control. Cells were stained for cilium (anti-Ac.Tub, red), GFP-Gli2 (anti-GFP, green) and nucleus (DAPI, blue). The same pictures are also shown with the channel corresponding to GFP-Gli2 in grey scale to help the visualization. Scale bar: 10 μm. (C) Quantification of nuclear Gli2 was performed as explained in Fig 1C. Black, solid line indicates comparison between control and Hh activated conditions for wtGli2, dotted line indicates comparison between wtGli2 under basal condition and mutNLS-1 or mutNLS-1+2 under basal or activated conditions and black, discontinued line indicates comparison between activated conditions for wtGl2 and mutNLS-2. *p<0.01, *** p<0.0001 (Kruskal-Wallis test). (B) and (C) are representative of four independent experiments and at least 70 cells were analysed for each condition. (D) WB detecting Imp-α1 after precipitating GFP-Gli2 or GFP-Gli2-mutNLS-1+2 with GFP-Trap from NIH/3T3 Flp-In expressing at endogenous levels the constructs mentioned above. Membranes were cut at different levels so as to detect in the same samples the precipitated GFP-Gli2 (using an anti-Gli2 antibody) and GFP (left panels).

To test whether the Gli2 movements into the cilium and the nucleus have mechanisms in common, we first studied Gli2 nuclear import, a process that has not been addressed using the entire Gli2 protein. Sequence analysis of mammalian Gli proteins and Cubitus interruptus (Ci), the *Drosophila melanogaster* orthologous, predicted two conserved cNLS, cNLS1 and cNLS2, expected to bind the Imp-α/β1 complex [29,40] (Fig 1A). In the case of Gli1, the involvement of Imp-β1 in nuclear import was first inferred by observing that mutations in these cNLSs inhibit the accumulation of Gli1 in the nucleus [29]. More recently, Szczepny *et al*. (2014) verified experimentally that Imp-β1 binds to both cNLS in Gli1 and mediates the nuclear import of a truncated form of the protein that contains these two regions [41]. Thus, we first verified the involvement of Imp-β1 in Gli2 nuclear import by using importazole (IPZ), a specific inhibitor of Imp-β1 mediated transport [42]. We transduced NIH/3T3 cells with lentivirus coding for GFP-Gli2, selected cells for stable expression of the protein, and studied GFP-Gli2 subcellular distribution by immunofluorescence and confocal microscopy. We observed that IPZ, in conditions where it inhibited Ca^+2^-induced translocation of the known Imp-β1 cargo NFAT [35] (S1A and S1B Fig), completely inhibited Gli2 nuclear accumulation after Hh pathway activation with SAG, a Smo agonist [32] (Fig 1B and 1C). Consistent with decreased Gli2 nuclear localization we observed that IPZ also inhibited the SAG-induced expression of a luciferase-based Hh reporter gene (Fig 1D). IPZ did not change the total levels of GFP-Gli2 (Fig 1E), indicating that the observed results must be the consequence of the inhibition of nuclear Imp-β1-mediated import. In order to verify the physical interaction between Gli2 and the Imp-α/β1 complex we performed immunoprecipitation of GFP-Gli2 from HEK293FT cells transfected with pEGFP-Gli2 and observed that both Imps interacted with Gli2 (Fig 1F and 1G). We then studied which of the two cNLS participate in moving Gli2 into the nucleus. We mutagenized the Gli2 cNLSs by introducing the same mutations that were previously tested in Gli1 [29], either separately (mutNLS-1, mutNLS-2) or together (mutNLS-1+2). In each case, the mutation abolished the prediction of the corresponding sequence as a cNLS (Fig 2A). We obtained stable cell lines expressing the mutant forms of GFP-Gli2 and analysed the localization of GFP-Gli2 in the nuclear compartment after stimulation of the Hh pathway with SAG. We observed an increase in Gli2 nuclear fluorescence only in the case of the wt protein (Fig 2B and 2C). Mutation of any of the Gli2 cNLS impaired SAG-induced Gli2 nuclear import, the effect being more pronounced when both regions were mutated (Fig 2B and 2C), suggesting that both regions play a functional role in Gli2 nuclear import. A result that caught our attention was that in the absence of pathway activators, whereas mutNLS-1 had the expected effect in Gli2 nuclear localization, NLS-2 had no effect (Fig 2B and 2C), suggesting that under basal conditions this NLS-2 may be hidden. Consistent with Imp α/β1 binding to these regions, the interaction of GFP-Gli2-mutNLS-1+2 with Imp-α was reduced compared to the wt protein (Fig 2D).

### Gli2 cNLSs are not required for the active transport of Gli2 into the cilium

**Fig 3:**
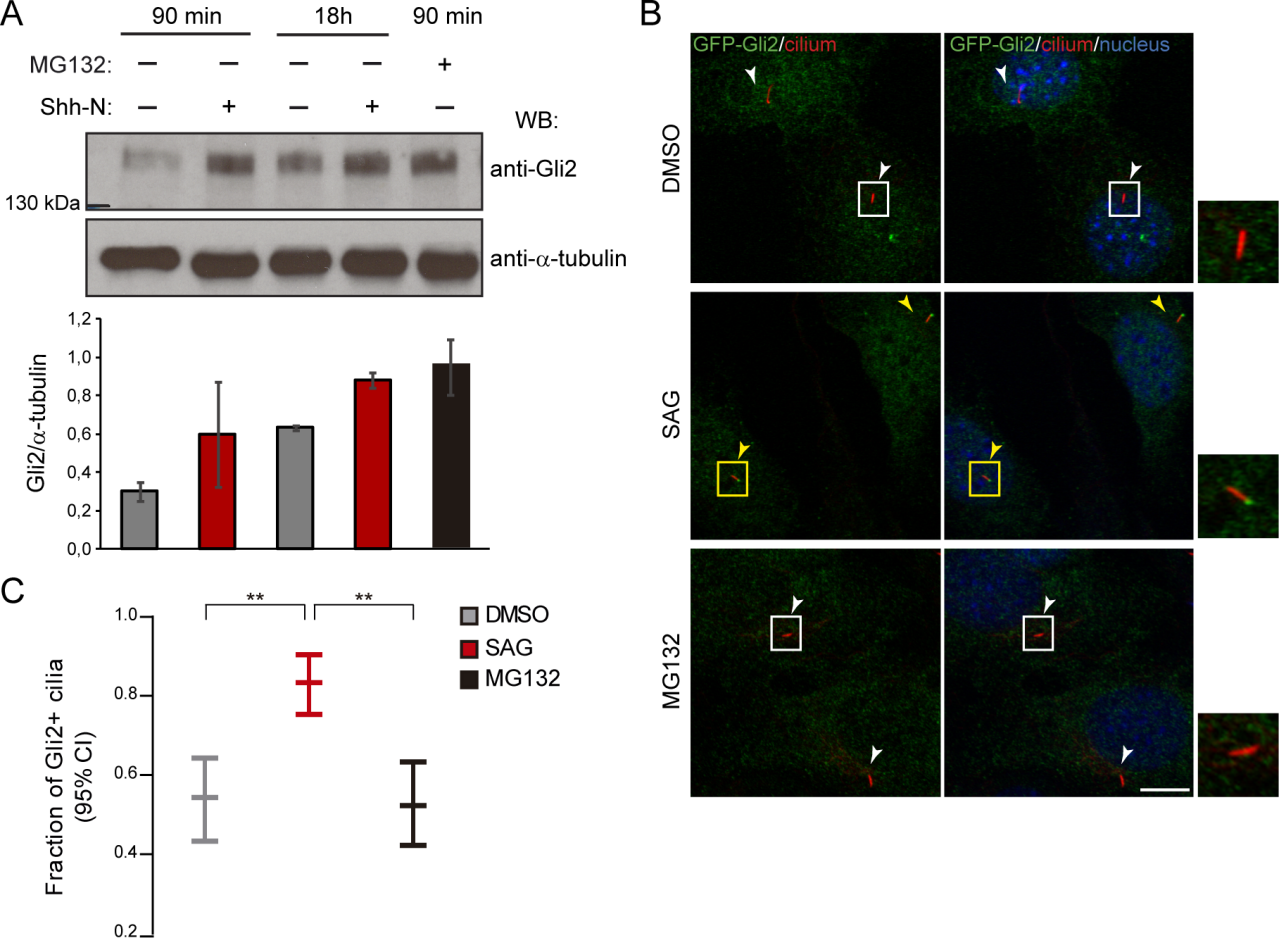
The accumulation of Gli2 at the ciliary tip is not just a consequence of increased proteins levels, but requires activation of the Hh pathway. (A) WB showing Gli2 levels in control cells, cells stimulated with Shh-N for 90 min or 18 h and cells treated with the proteasome inhibitor MG132 for 90 min. Gli2 levels were normalized against α-tubulin and the graph shows mean ± s.d of biological duplicates. (B) Analysis of Gli2 ciliary localization in transduced NIH/3T3 expressing GFP-Gli2 treated as described in (A). Cells were stained for cilium (anti-Ac.Tub, red), GFP-Gli2 (anti-GFP, green) and nucleus (TOPRO, blue). Small pictures show amplification of the selected region. Yellow and white arrows indicate cilia with or without Gli2 at the ciliary tip respectively. Scale bar: 10 μm. (C) Quantification of Gli2 ciliary localization. Results are expressed as the fraction of cilia that showed Gli2 staining (Gli2+ cilia) with 95% confidence interval (CI). At least 85 cilia were analysed for each sample. ** p<0.001 (hypothesis test for proportions).

The movement of Gli2 to the nucleus and the expression of Hh target genes are the last steps in pathway transduction. Before this, activation of Hh signalling leads to the inhibition of PKA and of proteasome-mediated degradation of Gli2 [27,43], and to the recruitment of Gli2 to the cilium [23,31]. Thus, we first decided to evaluate the formal possibility of Gli2 accumulating in the cilium simply as a consequence of increased overall Gli2 levels due to the inhibition of its degradation. To test this we inhibited the proteasome in non-stimulated cells and analysed Gli2 ciliary localization. Importantly, although proteasomal inhibition yielded Gli2 levels that were comparable to those of Hh-stimulated cells (Fig 3A), we did not observe the increase in Gli2 positive cilia that occurs in Hh stimulated cells (Fig 3B and 3C). Thus, this result supports the prevailing notion that an active transport mechanism is involved in the translocation of Gli2 into the ciliary compartment.

**Fig 4:**
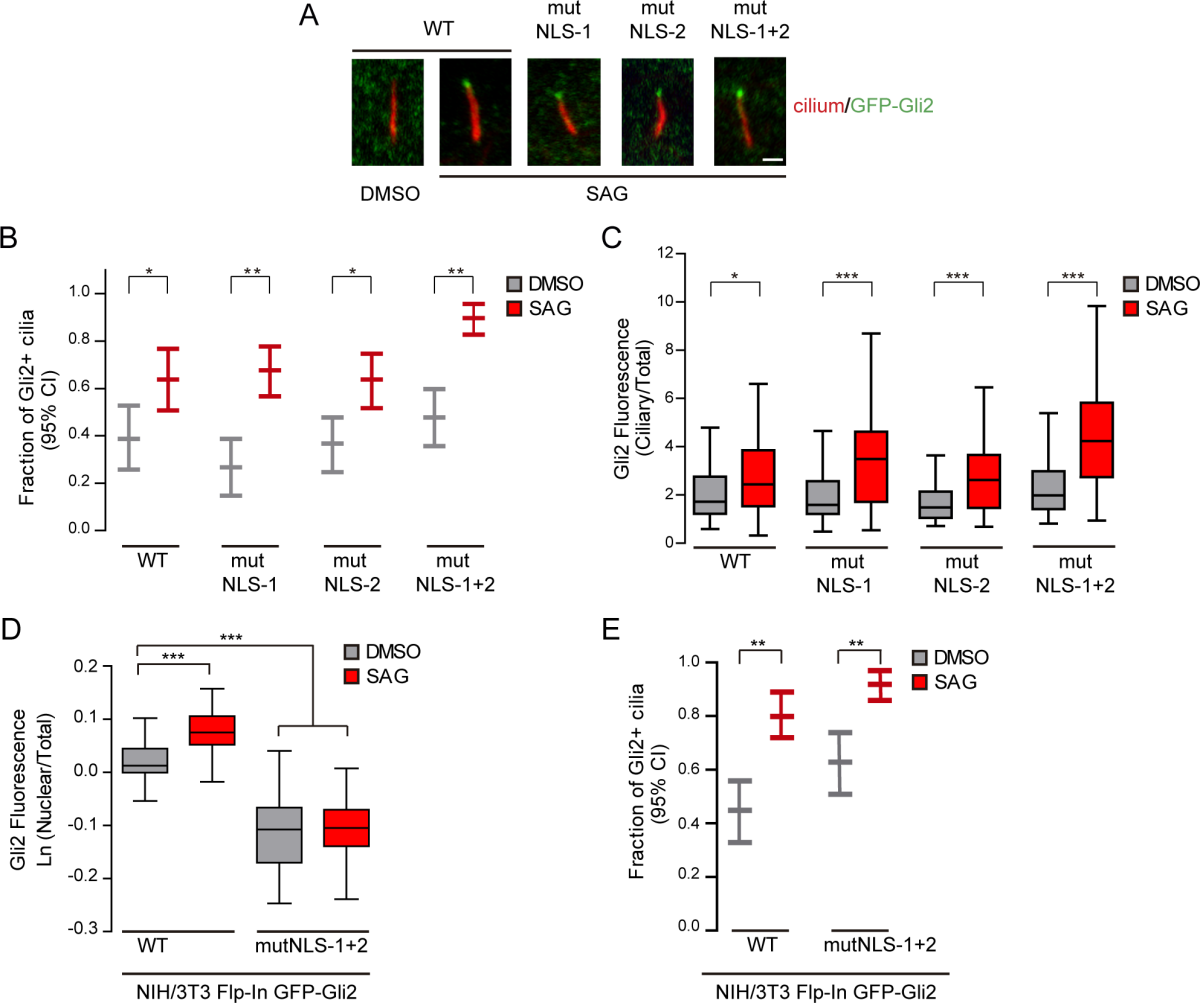
None of the two cNLS are necessary for Gli2 ciliary traffic. (A) NIH/3T3 cells transduced for expressing GFP-Gli2, wt or mutants in cNLS (mutNLS-1, mutNLS-2 or mutNLS-1+2) were treated with SAG for 90 min or DMSO as control. Cells were stained for cilium (anti-Ac.Tub, red) and GFP-Gli2 (anti-GFP, green). Images show representative cilia from each condition. Scale bar: 1 μm. (B) Quantification of Gli2 ciliary localization. Results are expressed as the fraction of Gli2+ cilia with 95% CI. At least 55 ciliated cells were analysed for each condition. *p<0.05, ** p<0.001 (hypothesis test for proportions). (C) The amount of ciliary Gli2 was estimated measuring the GFP fluorescence at the ciliary tip as explained in Materials and Methods. The mean ciliary fluorescence was normalised against the mean total GFP fluorescence of the cell, so as to correct for variations in Gli2 expression among different cells.* p<0.05, *** p<0.0001 (Kruskal Wallis test). (A-C) are representative of four independent experiments. (D-E) NIH/3T3 Flp-In stable cell lines expressing GFP-Gli2 or GFP-Gli2-mutNLS-1+2 were treated with SAG or DMSO as control in the same conditions than those described in (A) and cells were analyzed by confocal microscopy. The amount of nuclear Gli2 (D) was estimated as described in legend of Fig 1C. At least 60 cells were analysed for each condition. *** p<0.0001 (ANOVA). The fraction of Gli2 positive cilia (E) was quantified as described in part (B) of this legend. ** p<0.001 (hypothesis test for proportions).

We next studied the role of the Gli2 cNLSs in ciliary traffic. We analysed the capacity of -Gli2-mutNLS to move to the cilium upon activation of the Hh pathway with SAG. We used the transduced cells expressing our constructs and observed that both the percentage of Gli2 positive cilia (Fig 4A and 4B) and the amount of ciliary Gli2 (Fig 4C) in cells expressing single and double Gli2-mutNLS were comparable to those of cells expressing wtGli2, indicating that these cNLSs are not required for Gli2 ciliary movement. We did not have evidences of Gli2 overexpression in transduced cells but to rule out the possibility that Gli2-mutNLS was trafficking to the cilium as a consequence of being overexpressed, we obtained stable cell lines expressing GFP-Gli2 or GFP-Gli2-mutNLS-1+2 at the same levels than the endogenous Gli2. These cells were constructed using the Flp-In system (Life), in which the expression construct is introduced as a single-copy insertion into a defined locus in the genome by Flp-mediated recombination (see Materials and Methods). This system showed several advantages in comparison with the cell lines obtained by lentiviral transduction. First, all cells express the construct and at levels that are comparable to those of endogenous Gli2 (S2 Fig) [38]. Moreover, the construct is expressed under the EF1-α promoter, which is not silenced in NIH/3T3 cells, contrary to what happens with the CMV promoter used in our lentiviral system, thus enabling us to maintain the cell line without losing expression of the gene. Importantly, using these cells we obtained exactly the same results than using transduced cells: cNLSs, in spite of mediating Gli2 nuclear import (Fig 4D) are dispensable for Gli2 ciliary transport (Fig 4E). Given that mutation of the two cNLSs decreased the binding of Gli2 to Imp-α (Fig 2D) without affecting Gli2 ciliary localization, these results indicate that Imp-α/β1 is not involved in Gli2 ciliary traffic.

### A constitutively active form of Ran inhibits Gli2 movement to the cilium

To continue testing whether other components of the nuclear import machinery could play a role in Gli2 ciliary entry we next evaluated the involvement of the RanGTP/GDP gradient, a key player in importin-mediated transport. Importantly, it has been shown that RanGTP accumulates in the ciliary compartment as well as in the nucleus, and the RanGTP/GDP gradient is necessary for the movement of Kif17 into the cilium [15].

**Fig 5:**
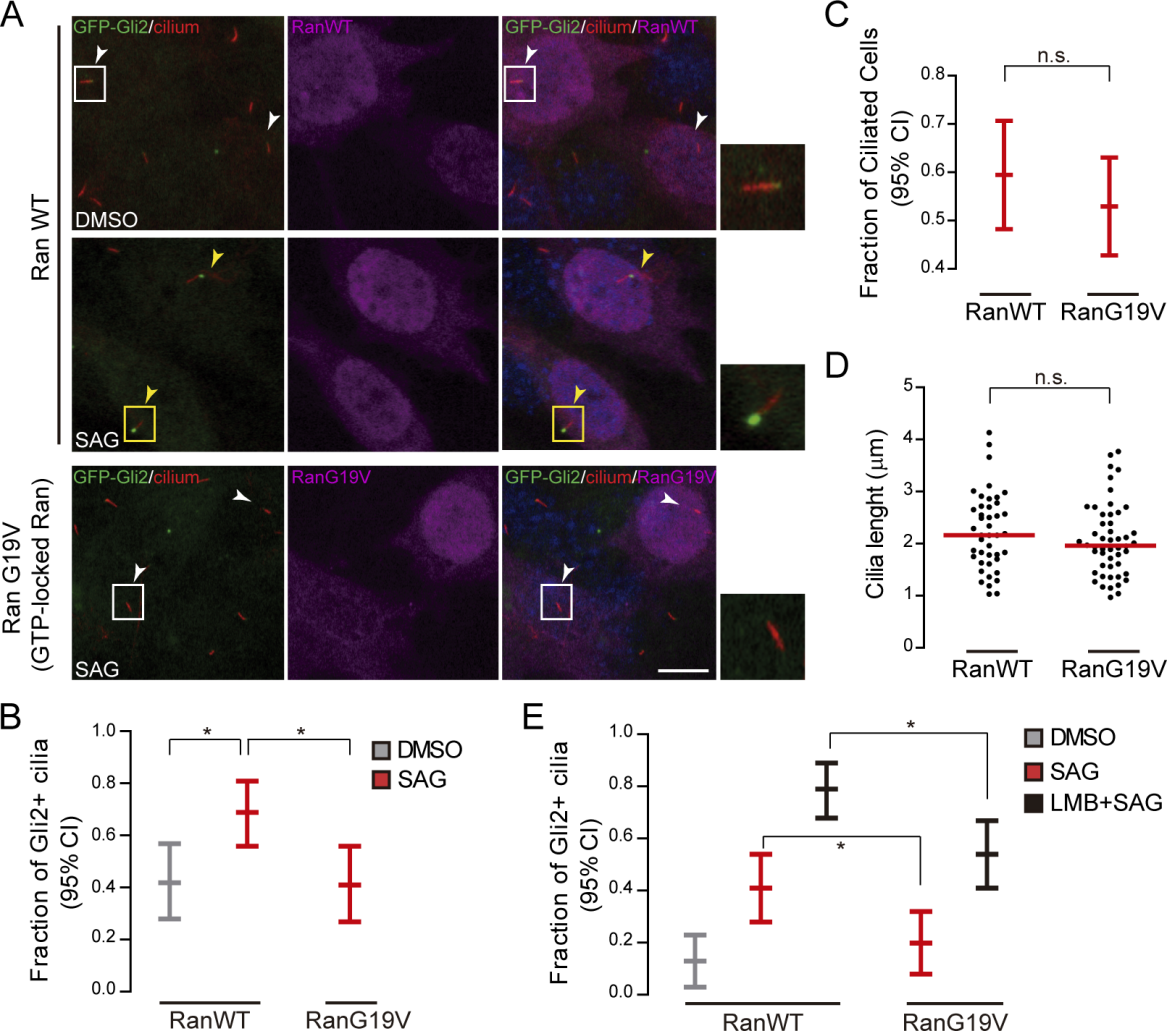
A GTP-locked form of Ran inhibits Gli2 ciliary localization. (A) NIH/3T3 Flp-In cells expressing GFP-Gli2 were transfected with either myc-RanWT or myc-RanG19V, stimulated with SAG or DMSO as a control, and Gli2 ciliary localization was determined in transfected cells. Cells were stained for cilium (anti-Ac.Tub, red), GFP-Gli2 (anti-GFP, green), myc-Ran (anti-myc, magenta) and nucleus (DAPI, blue). Small pictures show amplification of the selected region. Yellow and white arrows indicate cilia with or without Gli2 at the ciliary tip respectively. Scale bar: 10 μm. (B) Quantification of Gli2 ciliary localization in transfected cells. Results are expressed as the fraction of Gli2+ cilia in transfected cells with 95% CI. At least 50 cilia from transfected cells were analysed for each sample. * p<0.01 (hypothesis test for proportions). (C) Proportion of ciliated cells among transfected cells, expressed as 95% CI. At least 70 transfected cells were analysed in each condition. n.s. (not significant) p>0.05 (hypothesis test for proportions).(D) Measurement of cilia length in transfected cells. Each point represents a measurement for a single cilium; red lines represent the median length. At least 50 cilia were measured for each condition. n.s. (not significant) p>0.05 (Mann-Whitney test). (A-D) are representative of three independent experiments. (E) We analysed if LMB could revert the inhibition on Gli2 ciliary localization produced by RanG19V, by repeating the experiment showed in A but activating the Hh pathway in the presence of LMB. Results are expressed as in B. At least 50 cilia were analysed for each condition. * p<0.05 (hypothesis test for proportions).

We transfected NIH/3T3 Flp-In GFP-Gli2 cells with a plasmid coding for myc-tagged Ran proteins, WT or a constitutively active, GTP-bound, G19V mutant [44]. This mutant form of Ran will remain bound to Imps thus inhibiting the capacity of transporters to bind cargoes. We stimulated cells with SAG and determined the percentage of Gli2 positive cilia in Ran-transfected cells. Similarly to what was described for Kif17, expression of RanG19V completely inhibited the SAG-induced increase in Gli2 positive cilia that was observed in cells expressing RanWT (Fig 5A and 5B). Importantly, overexpression of RanG19V in our experimental setting (confluent cells cultured in 0.5% of CBS) did not affect either the percentage of ciliated cells or cilia length (Fig 5C and 5D). We obtained the same results when we analysed the localization of endogenous Gli2 in NIH/3T3 cells using a specific antibody, and stimulated the pathway with Shh-N-conditioned medium for 18 hours (S3 Fig). Thus, our results indicate that a central component of the nuclear-cytoplasmic shuttling machinery, the RanGTP/GDP gradient, is involved in Gli2 ciliary traffic.

Santos and Reiter (2014) suggested that the exportin CRM1 is involved in the exit of Gli2 from the cilium as leptomycin B (LMB), a specific inhibitor of CRM1 [45], induced the accumulation of Gli2 in the cilium [46]. As exportins bind cargoes only in the presence of RanGTP [47], overexpression of a GTP-locked Ran could favour their activity, as has been described for the export of several nuclear proteins [48]. Therefore, the inhibition of Gli2 ciliary localization that we observed in the presence of RanG19V could be the consequence of an impaired Gli2 ciliary entry or alternatively, an increased Gli2 ciliary exit. In order to discriminate between these two possibilities, we repeated the previous experiment in the presence of LMB, thus impairing CRM1-dependent export. As expected, LMB treatment increased the amount of nuclear Gli2 (S4 Fig) and also the percentage of Gli2 positive cilia, both in non-stimulated and SAG-treated cells (Fig 5E), confirming that Gli2 is actively exported out of the nucleus and the cilium by a LMB sensitive, and likely CRM1-dependent, mechanism. Importantly however, LMB did not restore the levels of Gli2 positive cilia in RanG19V expressing cells to those of control, RanWT expressing cells, indicating that RanG19V is affecting the entry of Gli2 into the cilium.

### Imp-β2 is involved in Gli2 ciliary transport

**Fig 6:**
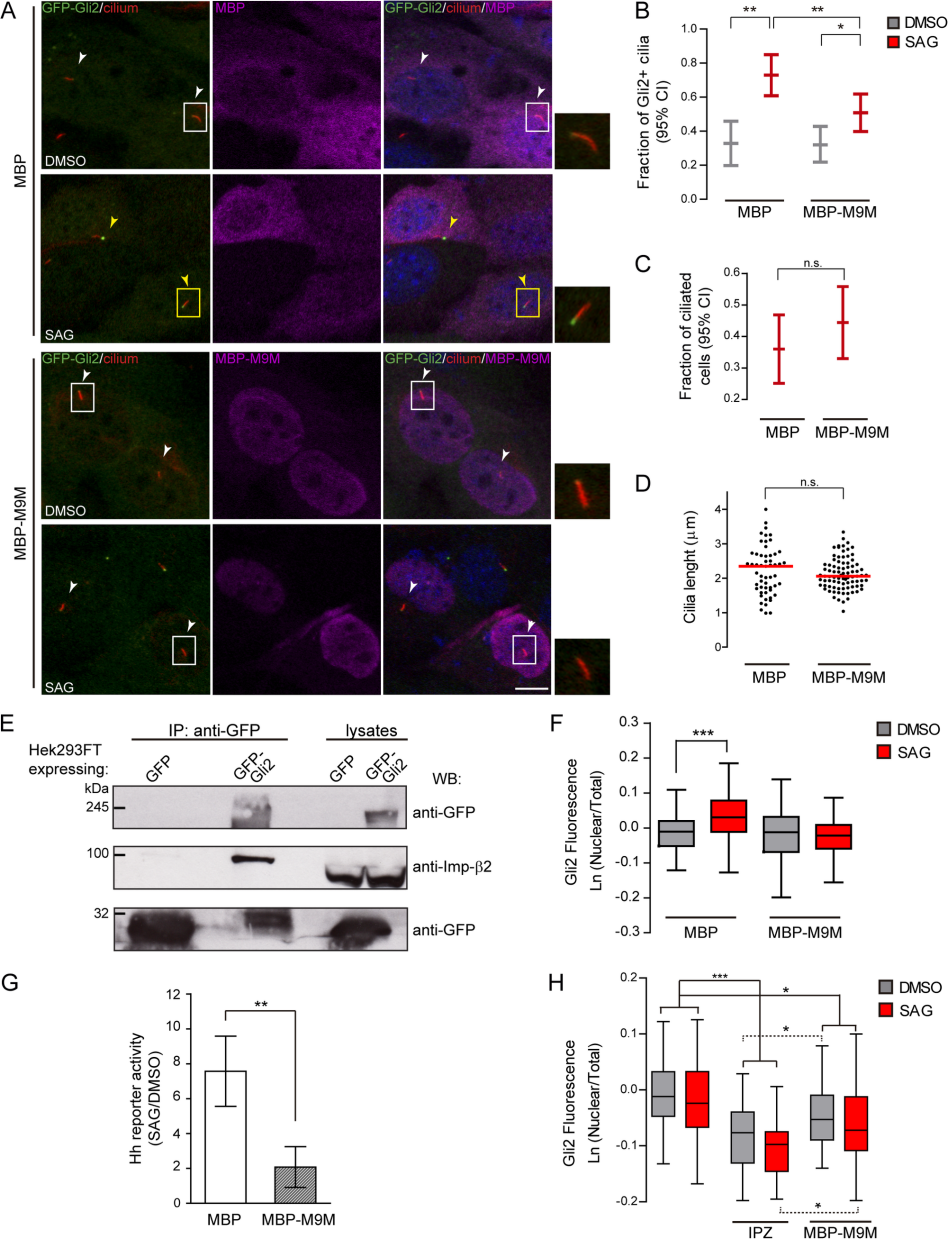
Imp-β2 is involved in moving Gli2 into the cilium. (A) NIH/3T3 Flp-In cells expressing GFP-Gli2 were transfected with either myc-MBP-M9M or myc-MBP as control, stimulated with SAG and Gli2 ciliary localization was analysed in transfected cells. Cells were stained for cilium (anti-Ac.Tub, red), GFP-Gli2 (anti-GFP, green), myc-MBP or myc-MBP-M9M (anti-myc, magenta) and nucleus (DAPI, blue). Small pictures show amplification of the selected region. Yellow and white arrows indicate cilia with or without Gli2 at the ciliary tip respectively. Scale bar: 10 μm. (B) Quantification of Gli2 ciliary localization in transfected cells. Results are expressed as the fraction of Gli2 positive cilia in transfected cells with 95% CI. At least 50 cilia from transfected cells were analysed for each sample.* p<0.05, **p<0,001 (hypothesis test for proportions). (C) Fraction of ciliated cells among transfected cells, expressed as 95% CI. At least 120 transfected cells were analysed in each condition. n.s. (not significant) p>0.05 (hypothesis test for proportions). (D) Measurement of cilia length in transfected cells. Each point represents a measurement for a single cilium; red lines represent the median length. At least 60 cilia were measured for each condition. n.s. (not significant) p>0.05 (Mann-Whitney test). (E) WB detecting Imp-β2 after precipitating GFP-Gli2 with GFP-Trap from HEK293FT transfected with pEGFP-Gli2. Membranes were cut at different levels so as to detect in the same samples the precipitated GFP-Gli2 and GFP. The levels of GFP-Gli2, Imp-β2 and GFP in the lysates used for immunoprecipitation were assessed by WB. The band corresponding to GFP looks distorted because the protein migrates with the dye front. (F) Quantification of nuclear Gli2 in transfected cells was performed as described in Fig 1C. At least 60 cells were analysed for each condition. *** p<0.0001 (Kruskal-Wallis test). (A-E) are representative of 3 independent experiments. (G) Activation of a luciferase-based Hh reporter gene in NIH/3T3 transfected with plasmids coding for myc-MBP or myc-MBP-M9M and then stimulated with SAG or DMSO as control. As explained in Materials and Methods, RLU values from SAG treated cells are normalised against the RLU values from non-activated cells and expressed as mean ± s.d from triplicates from two independent experiments. ** p<0.001 (Mann-Whitney test). (H) Quantification of nuclear Gli2 in NIH/3T3 cells transfected with pEGFP-Gli2 alone, or pEGFP-Gli2 plus plasmids coding for myc-MBP or myc-MBP-M9M. Some cells transfected with pEGFP-Gli2 alone were treated with IPZ for 1 hour before activation of the Hh pathway with SAG. In the case of cells tranfected with myc-MBP or myc-MBP-M9M, Gli2 nuclear fluorescence was determined in myc-positive cells. Quantification was performed as described in legend to Fig 1C. Results are representative of two experiments and at least 50 cells were analysed for each condition.* p<0.05, *** p<0.0001 (ANOVA).

The identification of a critical role for the RanGTP gradient in Gli2 ciliary entrance suggested that Imps could participate in the process. Therefore, we studied the involvement of Imp-β2 in Gli2 ciliary transport, as this transporter is responsible for the movement of other ciliary proteins [15,16]. We transfected NIH/3T3 Flp-In GFP-Gli2 expressing cells with plasmids coding for MBP fused to the peptide M9M or MBP alone as a control. M9M is a 37 amino acids-long peptide designed by fusing the hot spots of two PY-NLS from prototype Imp-β2 cargoes, namely hnRNPA1 and hnRNPM. M9M binds to the PY-NLS binding site of Imp-β2 200-fold tighter than a wt PY-NLS, acting as a potent and specific Imp-β2 inhibitor [49]. As expected, in cells expressing MBP-M9M, nuclear import of hnRNPA1 was decreased while NFAT (an Imp-β1 cargo) accumulation was unaffected in comparison with cells that expressed MBP alone, hence confirming the specificity of the assay (S5 Fig). We then assessed Gli2 ciliary localization upon MBP and MBP-M9M transfection. While the percentage of ciliated cells and cilia length were not altered (Fig 6C and 6D), M9M significantly diminished the SAG-induced increase in the percentage of Gli2 positive cilia in comparison with cells transfected with MBP alone (Fig 6A and 6B). Thus, our results indicate that Imp-β2 is involved in Gli2 movement into the cilium. We studied the ability of Gli2 to interact with Imp-β2 by performing an immunoprecipitation from HEK293FT cells transfected with pEGFP-Gli2 and confirmed the interaction between the two proteins (Fig 6E). Moreover, different to what was observed in MBP-expressing cells, those expressing M9M did not accumulate Gli2 in the nucleus after Hh activation (Fig 6F) and consequently showed reduced activity of the pathway in a luciferase-based Hh reporter assay (Fig 6G). Though this is consistent with previous reports that showed that Gli2 has to enter the cilium in order to dissociate from SuFu and be then imported into the nucleus [50], we could not rule out the possibility that Imp-β2 may additionally be involved in moving Gli2 into the nucleus, as was described for Ci [51]. In order to evaluate the latter possibility we activated the Hh pathway bypassing the cilium by Gli2 overexpression. This procedure has been shown to activate the Hh pathway in the absence of ligand and independently of the cilium [52,53]. Thus, we transfected cells with pEGFP-Gli2 and analysed Gli2 nuclear localization in the presence or absence of M9M. In parallel, we treated cells with IPZ, to verify that under these conditions nuclear import could be inhibited. As expected, Gli2 overexpression in the absence of Hh pathway agonists resulted in levels of nuclear Gli2 equal to those induced by SAG stimulation (Fig 6H). In these conditions IPZ was able to reduce the amount of nuclear Gli2 (Fig 6H), reinforcing our previous results on the involvement of Imp α/β1 in Gli2 nuclear import. M9M, less potently than IPZ, also reduced the level of nuclear Gli2, suggesting that Imp-β2, in addition to move Gli2 into the cilium, may be involved in Gli2 nuclear import.

In summary, our work shows that the nuclear import machinery participates in moving Gli2 into the cilium. Though similar results were obtained with other ciliary proteins [15,16] it is the first time that this mechanism is involved in the ciliary traffic of a protein that can also localize in the nucleus. In this case, the main transporters involved in nuclear and ciliary import are not the same: the Imp-α/β1 complex binding to cNLS are involved in Gli2 nuclear import but not in the trafficking to the cilium, this last process being mediated instead by Imp-β2.

## Discussion

Compartmentalization has provided eukaryotic cells with an efficient mechanism of regulating different cellular processes. The nucleus and cilium are good examples of this strategy, as highlighted by the Hh pathway. Regulation by differential localization relies on efficient mechanisms of protein targeting and transport to specific cellular destinations. In contrast to other cellular organelles, the nuclear and ciliary compartments are topologically equivalent to the cytosol; however, mechanisms exist that assure their specific protein composition. Interestingly, in the last couple of years several similarities have been described between the transport of soluble proteins into the nucleus and the cilium (reviewed in references [9,54]). In this work we have chosen Gli2 to study if these similarities also apply to a protein that can localize to both compartments.

Gli2 is the main transcriptional activator of the Hh pathway in mammals. Its activity is regulated by different mechanisms, compartmentalization being one of them. In the absence of Hh, Gli2 is mainly retained in the cytosol and degraded. Upon Hh signalling, Gli2 moves into the cilium where it dissociates from SuFu and is then able to reach the nucleus. We have focused on these two events of protein transport in an effort to identify the mechanisms involved. We observed that Gli2 movement into the nucleus is mediated by the Imp-α/β1 complex through two Gli2 cNLSs that are conserved in other Gli proteins and Ci: mutating the two cNLSs or inhibiting Imp-β1 using IPZ led to decreased accumulation of Gli2 in the nucleus after Hh activation. These results are similar to those reported for Gli1 [29,41] but is the first time they are described using the entire Gli2 protein. In addition, Shi *et al*. (2014) have proposed that Ci and Gli2 can move into the nucleus using Imp-β2 through the interaction of this transporter with a region that resembles a PY-NLS (in Gli2_*Mm*, amino acids 201–247; PY-NLS_201-247_) [51]. They showed that this region interacts with purified Imp-β2 and is able to act as a NLS when fused to a carrier protein [51]. In agreement with these reports, we demonstrated that when Gli2 is overexpressed in order to bypass cilium regulation, the nuclear import of the protein can be partially blocked using the M9M peptide, a specific Imp-β2 inhibitor. However, it is possible to speculate that Imp-β2 might not be the main Gli2 nuclear transporter given that: i) when looking at cilia-independent Gli2 nuclear transport the inhibition produced by M9M was less pronounced than that resulting from blocking Imp-β1 with IPZ, and ii) when Gli2 was expressed at endogenous levels we did not observe IPZ-resistant Gli2 nuclear import after Hh activation. Finally, it is interesting to note that PY-NLS_201-247_ contains the cNLS-1, capable of binding the Imp-α/β1 complex [41]. Therefore, it is possible that Imp-α/β1 and Imp-β2 may compete for this region and thus further work is needed to determine which of the two binds to it under physiological conditions.

Regulation of Hh signalling must assure that Gli nuclear import occurs only after pathway activation. Thus, masking the cNLSs in the absence of signalling appears as an efficient strategy that in fact has been proposed for two inhibitors of the pathway, PKA [55] and SuFu [41], both of which inhibit Gli nuclear localization. The PKA-mediated phosphorylation of a threonine residue adjacent to cNLS-2 was proposed to inhibit Imp-β1 binding to Gli1[55]. As this Thr residue is conserved among the different Gli proteins (in Gli2_*Mm*, Thr556), it may constitute a general mechanism for silencing this cNLS. However, mass spectrometry analysis did not reveal this Thr as a phosphorylated residue in Gli2 [38]. Moreover, the inhibitory activity of SuFu is in part due to its ability to keep Gli proteins in the cytosol. SuFu binds to a region that contains a SYGH motif that is conserved among Gli proteins [56]. Binding of SuFu to this region has been shown to compete with Imp-β1 for binding to Gli1 cNLS-1 [41]. In a simplified scenario, activation of the Hh pathway results in the inhibition of PKA-mediated Gli2 phosphorylation [38], which could lead to the unmasking of NLS-2. Moreover, Gli traffic into the cilium, which results in disassembly of the Gli-SuFu complex, could expose cNLS-1 allowing nuclear translocation. It is interesting to note that analysing the Gli2 sequence with GloPlot [57] and DisEMBL [58] we observed that both cNLSs lie in, or near, regions predicted as highly disordered. This property could facilitate such masking/unfolding possibilities as has been described for other proteins [59].

Mutations in the two cNLSs that decreased the interaction with the Imp-α/β1 complex inhibited Gli2 nuclear import but did not affect the transport to the cilium, suggesting that Imp-α/β1 does not participate in this process. However, other reports support the idea that the nuclear import machinery could be involved in Gli2 ciliary traffic. It was shown that forced dimerization of NUP62, a nucleoporin present at the ciliary base, blocked the gated entry of Gli2, as well as several other cytosolic proteins into the cilium [14]. These experiments could not distinguish between physical impairment of protein movement from inhibition of a specific, Imp-mediated transport. By overexpressing a GTP-locked form of Ran, and therefore inhibiting binding of any Imp to their cargoes, we blocked Gli2 ciliary trafficking. In addition, by using a specific inhibitor we determined that Imp-β2 is involved in the process. Imp-β2 has been localized to the cilium by immunofluorescence [16] as well as by proteomic analysis [60]. How general or specific is the use of the nuclear-cytoplasmic shuttling machinery in the transport of proteins into the cilium is an open question whose answer should involve high-throughput approaches that are beyond the scope of this work. Having said that however, in our experimental setting where inhibition of this transport system was performed on ciliated cells, we did not observe significant changes in the percentage of ciliated cells or cilia length, suggesting that this system would not be required for the transport of proteins that are essential for the maintenance of the organelle.

Imp-β2 could bind Gli2 directly or through a Gli2 partner. In the first scenario we would expect Gli2 to have an Imp-β1 PY-NLS in its sequence, which could thus act as a ciliary localization signal (CLS). As mentioned above, one such motif has been proposed to be located between residues 201 and 247, PY-NLS_201-247_ [51]. However, its role as a CLS is not clear. For example, the two fragments that are generated after Gli2 proteolytic processing do not traffic to the cilium although the N-terminal one has the PY-NLS_201-247_ [46]. In contrast, a Gli2 protein lacking the repressor domain (Gli2Δ47–271) traffic to the cilium but less efficiently than the wt protein [46], thus suggesting that this region could contribute to the proposed CLS. Our data shows that the cNLS-1, which is included in the PY-NLS_201-247_ and thus absent in the Gli2Δ47–271 mutant, does not impair ciliary traffic. These data are not necessarily contradictory as it is difficult to compare the effect of our point mutations to that of a 224 amino acids-long deletion that might include additional important residues. In addition, the mutations that we introduced in this region to generate the mutNLS-1, were intended to disrupt the interaction with Imp-α/β1 and not with Imp-β2. In fact, it has been shown that changing only the amino acids that we targeted in our cNLS-1 mutant is not enough for abrogating Imp-β2 binding in some proteins [61,62]. Thus, it still cannot be determined whether Imp-β2 requires the PY-NLS_201-247_ for mediating Gli2 ciliary traffic.

Other groups that have looked for specific regions in Gli2 that can act as CLS showed that only large deletions (Δ851–1183 or Δ570–967, 331 and 397 amino acids long respectively) are able to abrogate its ciliary localization [46,63]. Although Liu and colleagues (2015) have recently shown that one of these mutants (Gli2Δ570–967) retains its transcriptional activity [50], it cannot be rule out that inhibition of ciliary localization is due to gross changes in Gli2 conformation rather than removal of a specific CLS. By following the rules described for the identification of potential PY-NLS [61], we were able to detect one additional motif (in Gli2_*Mm*, amino acids 800–822) that is located near the CLS described by Santos *et al*.[46] and within that described by Zheng *et al*. [63]. Further experimentation will be needed to test whether this region is a *bona fide* Imp-β2 binding sequence. Lastly, other regions of Gli2 that are not recognized as PY-NLSs could be involved in mediating the interaction with Imp-β2, as has been described for several nuclear Imp-β2 cargoes [12]. NLSs that act as CLS have been found in Kif17 [15] and RNP2 [16], other ciliary proteins transported by Imp-β2. It is important to note that in those cases, the sequences act as CLS only in the context of the native protein, and when fused to an unrelated protein they function as regular NLSs moving proteins into the nucleus. This clearly shows that binding to Imp-β2 is not enough for directing proteins into the cilium, and other regions of the protein, by still unknown mechanisms, determine the final ciliary localization.

The second possible scenario is that Gli2 ciliary movement involves an adaptor protein that is recognized by Imp-β2. Candidates for these potential Gli2 partners are Gli2 interacting proteins that co-localize with it in the cilium. SuFu, despite presenting these two characteristics, seems not to be the sought partner since SuFu does not localize to cilia in the absence of Gli proteins [31,63] and ciliary localization of Gli proteins is independent of SuFu [31,52]. A potential candidate for mediating Gli2-trafficking to the cilium is Kif7, which has been shown to localize at the ciliary tip and to be necessary for accumulation of Gli proteins in this location upon Shh stimulation [64,65]. However, a recent work shows that Kif7 is necessary only for the accumulation of Gli2 at the ciliary tip but not for Gli2 to go across the ciliary TZ [65]. Finally, the possibility that Kif17 is actually involved in Gli2 ciliary movement should be explored. Kif17 belongs to the kinesin-2 family and has been shown to function as a ciliary motor both in photoreceptors and mammalian olfactory sensory neurons [66,67]. Although Gli2 interacts with the heterotrimeric kinesin- 2 (KIF3 complex) [68] there are no reports of Kif17 interacting with Gli proteins yet. Finally, factors yet to be understood must ensure the temporal sequence of events: first the engagement of Imp-β2 for moving Gli2 into the cilium, followed by the binding of Imp-α/β1 for entering the nucleus. SuFu binding, with the consequent hiding of cNLSs may contribute to the establishment of this sequence. In the same line of reasoning, it would be critical to identify the factors that determine why Imp-β2 moves Gli2 into the cilium instead of to the nucleus. In summary, our data support the notion that the nuclear-cytoplasmic shuttling machinery is involved in moving Gli2 into the nucleus and the cilium, while localization of the protein in one compartment or the other likely depends on the type of Imp that is engaged.

## Supporting Information

S1 Fig IPZ inhibits Ca^+2^-induced NFAT nuclear translocation. (A) NFAT nuclear translocation in NIH/3T3 expressing GFP-NFAT was induced with ionomycin in presence or absence of IPZ. NFAT was visualized by GFP fluorescence (green) and nucleus was stained with TOPRO (blue). Scale bar: 10 μm. (B) Nuclear NFAT was quantified measuring the mean GFP fluorescence in the nuclear compartment as explained in Materials and Methods for Gli2. The mean nuclear fluorescence was normalized against the mean total GFP fluorescence of the cell, so as to correct for variations in protein expression among different cells. Results are expressed as box plots as described in Material and Methods. At least 40 transfected cells were quantified. * p<0.05, *** p<0,0001(Kruskal-Wallis test).
(TIF)

S2 Fig GFP-Gli2 is expressed at the same level than endogenous Gli2 in NIH/3T3 Flp-In GFP-Gli2 cell lines. Western blot analysis of total extracts from two NIH/3T3 Flp-In GFP-Gli2 clones. We used an anti-GFP antibody that recognized only the band corresponding to GFP-Gli2 and an anti-Gli2 antibody that detected both, endogenous Gli2 and GFP-Gli2. Both bands show similar intensities, suggesting that GFP-Gli2 is expressed at similar levels than the endogenous protein.
(TIF)

S3 Fig RanG19V blocks endogenous Gli2 ciliary entry in cells stimulated with Shh-N conditioned medium. This experiment is the same as the one shown in Fig 5 but detecting endogenous Gli2 and activating the Hh pathway for a longer time using Shh-N conditioned medium. (A) NIH/3T3 cells were transfected with plasmids coding for Cerulean-tagged versions of Ran (WT or G19V) and the Hh pathway was activated with Shh-N conditioned medium for 18 h. Transfected cells were identified by cerulean fluorescence (magenta) and Gli2 was detected using an anti-Gli2 antibody (green). Small pictures show amplification of the selected regions. Yellow and white arrows indicate cilia with or without Gli2 at the ciliary tip respectively. Scale bar: 10 μm. (B) Quantification of Gli2 ciliary localization in transfected cells. Results are expressed as the fraction of Gli2+ cilia in transfected cells with 95% CI. At least 100 cilia from transfected cells were analysed for each sample. ** p<0.001 (hypothesis test for proportions). (C) Proportion of ciliated cells among transfected cells, expressed as 95% CI. At least 150 transfected cells were analysed in each condition. n.s. (not significant) p>0.05 (hypothesis test for proportions). (D) Measurement of cilia length in transfected cells. Each point represents a measurement for a single cilium; red lines represent the median length. At least 30 cilia were measured for each condition. n.s. (not significant) p>0.05 (Mann-Whitney test). (A-D) are representative of three experiments.
(TIF)

S4 Fig LMB induce the accumulation of Gli2 in the nuclear compartment. Transduced NIH/3T3 cells expressing GFP-Gli2 were treated with SAG or DMSO for 90 min in the presence or absence of LMB. Nuclear Gli2 was studied by immunofluorescence and confocal microscopy and quantified as described in the legend of Fig 1. At least 60 cells were measured for each condition. *p<0.05, *** p< 0,0001 (ANOVA).
(TIF)

S5 Fig M9M blocks hnRNPA1 but not NFAT nuclear import. (A) NIH/3T3 cell were transfected with plasmids coding for Flag-hnRNPA1 and either myc-MBP or myc-MBP-M9M. Eighteen hours later cells were stained for Flag-hnRNPA1 (anti-Flag, green), myc-MBP or myc-MBP-M9M (anti-myc, magenta) and nucleus (DAPI, blue). Scale bar: 10 μm. (B) Nuclear hnRNPA1 in MBP/MBP-M9M expressing cells was quantified measuring mean fluorescence in the nuclear compartment as explained in Materials and Methods. At least 55 cells were analysed. * p<0,05 (Kruskal-Wallis test). (C) NIH/3T3 cell were transfected with plasmids coding for GFP-NFAT and either myc-MBP or myc-MBP-M9M. NFAT nuclear translocation was stimulated with inonomycin. Cells were stained for MBP or MBP-M9M (anti-myc, magenta) and nucleus (DAPI, blue) and NFAT was visualized by GFP fluorescence (green). Scale bar: 10 μm. (D) Nuclear NFAT in MBP/MBP-M9M expressing cells was quantified measuring the mean GFP fluorescence in the nuclear compartment as explained previously. The mean nuclear fluorescence was normalised against the mean total GFP fluorescence of the cell. At least 45 transfected cells were quantified. *** p<0.0001 (ANOVA).
(TIF)
