# Supplementary figures and images for: Ciliary Entry of the Hedgehog Transcriptional Activator Gli2 Is Mediated by the Nuclear Import Machinery but Differs from Nuclear Transport in Being Imp-α/β1-Independent
Source: PLoS One. 2016 Aug 31;11(8):e0162033. doi: 10.1371/journal.pone.0162033 (PMC5007031; doi:10.1371/journal.pone.0162033)

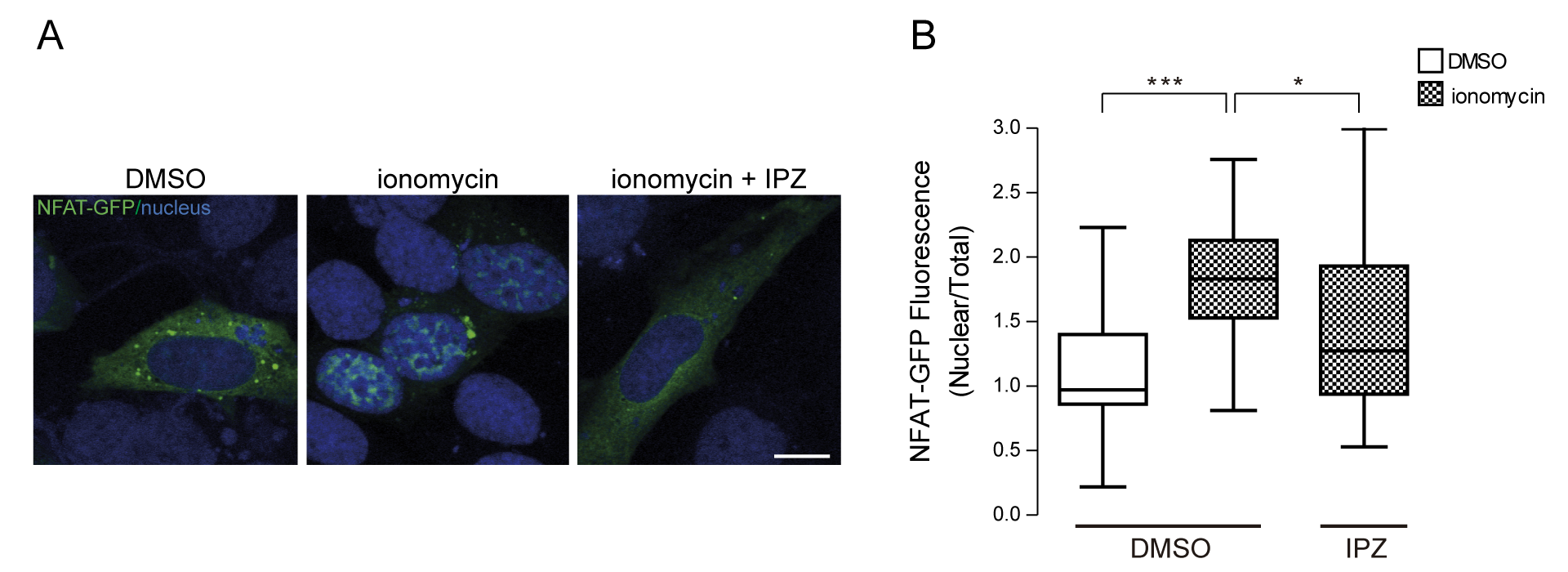

Supplement: S1 Fig — (A) NFAT nuclear translocation in NIH/3T3 expressing GFP-NFAT was induced with ionomycin in presence or absence of IPZ. NFAT was visualized by GFP fluorescence (green) and nucleus was stained with TOPRO (blue). Scale bar: 10 μm. (B) Nuclear NFAT was quantified measuring the mean GFP fluorescence in the nuclear compartment as explained in Materials and Methods for Gli2. The mean nuclear fluorescence was normalized against the mean total GFP fluorescence of the cell, so as to correct for variations in protein expression among different cells. Results are expressed as box plots as described in Material and Methods. At least 40 transfected cells were quantified. * p<0.05, *** p<0,0001(Kruskal-Wallis test). (TIF) [file pone.0162033.s001.tif]

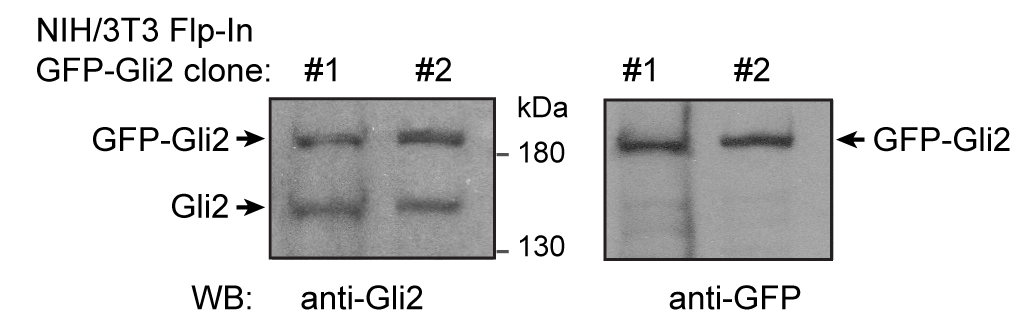

Supplement: S2 Fig — Western blot analysis of total extracts from two NIH/3T3 Flp-In GFP-Gli2 clones. We used an anti-GFP antibody that recognized only the band corresponding to GFP-Gli2 and an anti-Gli2 antibody that detected both, endogenous Gli2 and GFP-Gli2. Both bands show similar intensities, suggesting that GFP-Gli2 is expressed at similar levels than the endogenous protein. (TIF) [file pone.0162033.s002.tif]

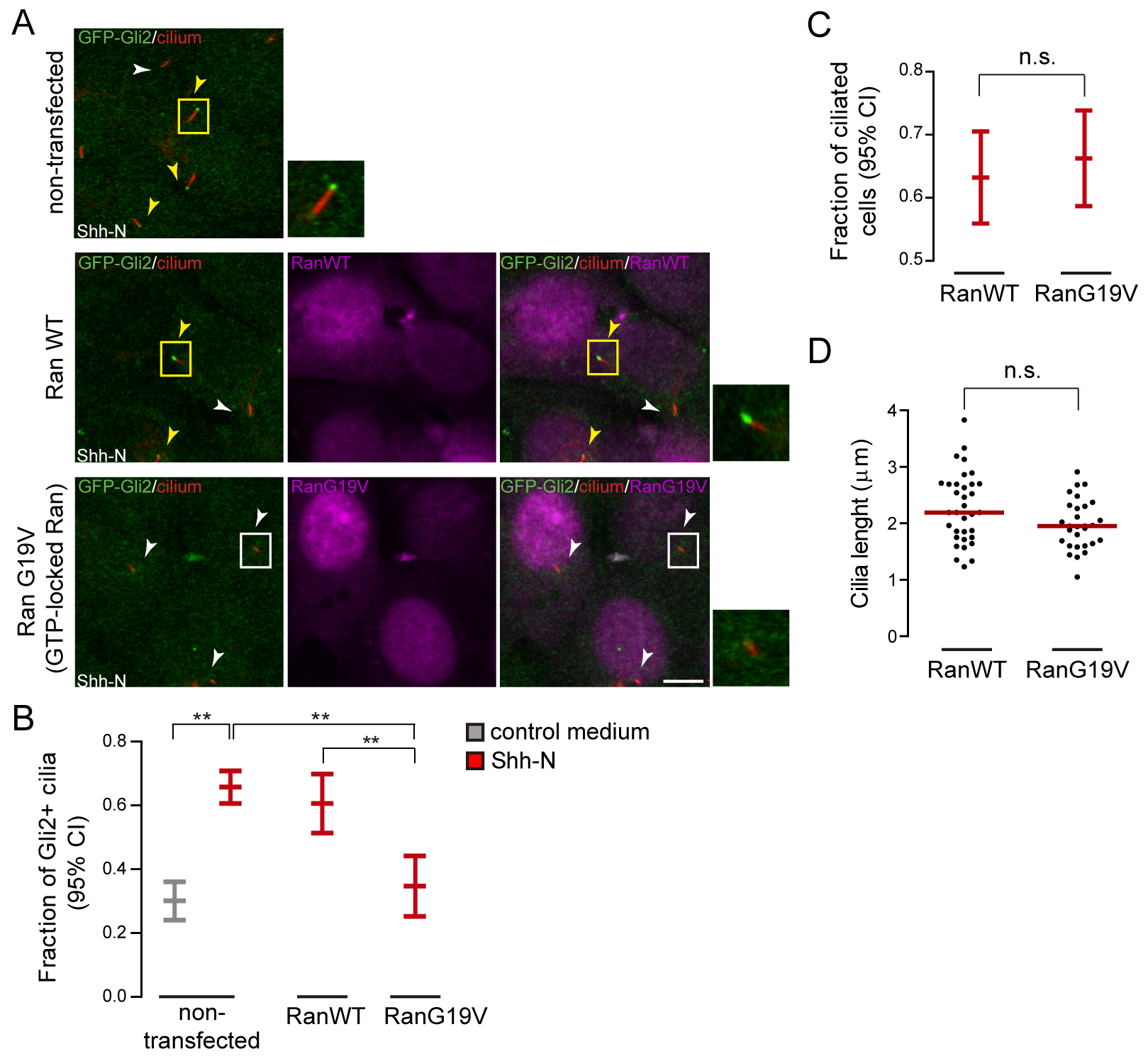

Supplement: S3 Fig — This experiment is the same as the one shown in Fig 5 but detecting endogenous Gli2 and activating the Hh pathway for a longer time using Shh-N conditioned medium. (A) NIH/3T3 cells were transfected with plasmids coding for Cerulean-tagged versions of Ran (WT or G19V) and the Hh pathway was activated with Shh-N conditioned medium for 18 h. Transfected cells were identified by cerulean fluorescence (magenta) and Gli2 was detected using an anti-Gli2 antibody (green). Small pictures show amplification of the selected regions. Yellow and white arrows indicate cilia with or without Gli2 at the ciliary tip respectively. Scale bar: 10 μm. (B) Quantification of Gli2 ciliary localization in transfected cells. Results are expressed as the fraction of Gli2+ cilia in transfected cells with 95% CI. At least 100 cilia from transfected cells were analysed for each sample. ** p<0.001 (hypothesis test for proportions). (C) Proportion of ciliated cells among transfected cells, expressed as 95% CI. At least 150 transfected cells were analysed in each condition. n.s. (not significant) p>0.05 (hypothesis test for proportions). (D) Measurement of cilia length in transfected cells. Each point represents a measurement for a single cilium; red lines represent the median length. At least 30 cilia were measured for each condition. n.s. (not significant) p>0.05 (Mann-Whitney test). (A-D) are representative of three experiments. (TIF) [file pone.0162033.s003.tif]

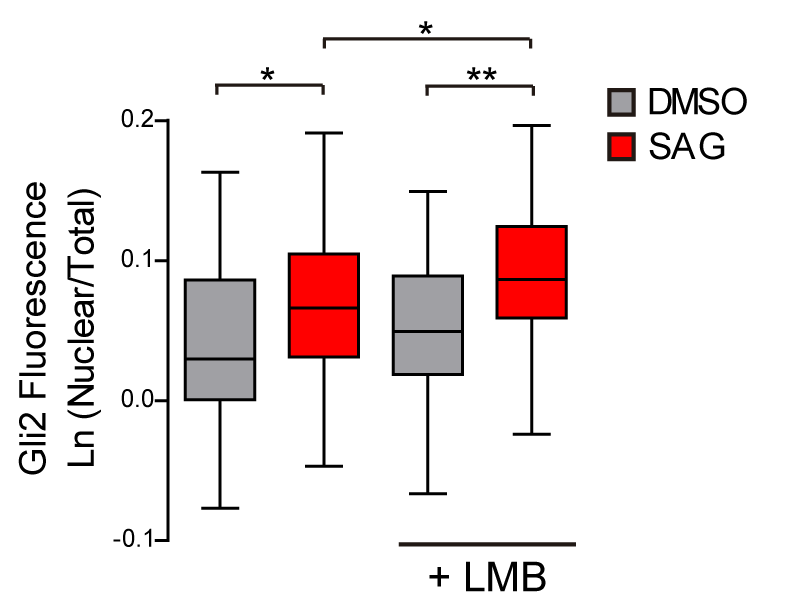

Supplement: S4 Fig — Transduced NIH/3T3 cells expressing GFP-Gli2 were treated with SAG or DMSO for 90 min in the presence or absence of LMB. Nuclear Gli2 was studied by immunofluorescence and confocal microscopy and quantified as described in the legend of Fig 1. At least 60 cells were measured for each condition. *p<0.05, *** p< 0,0001 (ANOVA). (TIF) [file pone.0162033.s004.tif]

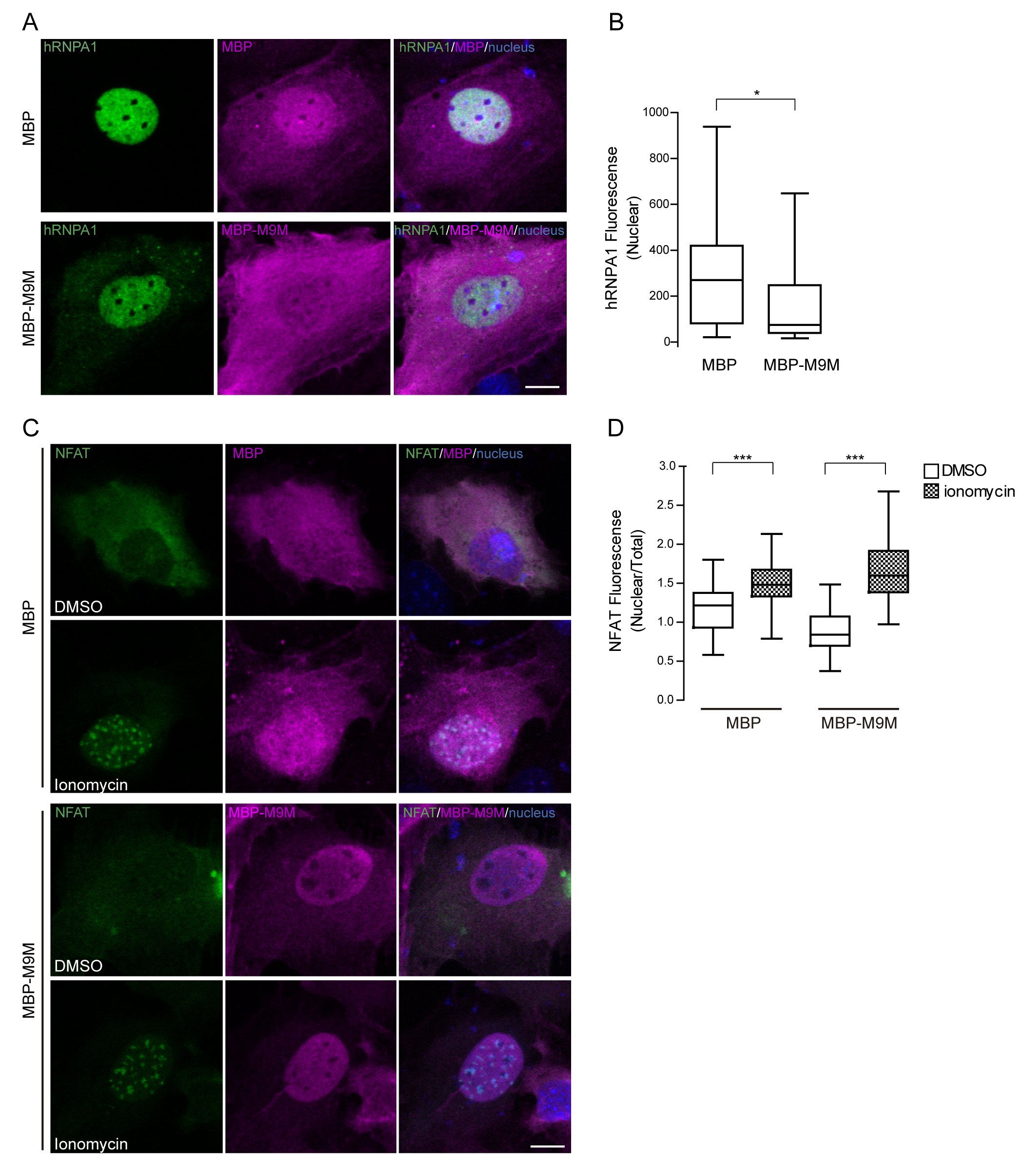

Supplement: S5 Fig — (A) NIH/3T3 cell were transfected with plasmids coding for Flag-hnRNPA1 and either myc-MBP or myc-MBP-M9M. Eighteen hours later cells were stained for Flag-hnRNPA1 (anti-Flag, green), myc-MBP or myc-MBP-M9M (anti-myc, magenta) and nucleus (DAPI, blue). Scale bar: 10 μm. (B) Nuclear hnRNPA1 in MBP/MBP-M9M expressing cells was quantified measuring mean fluorescence in the nuclear compartment as explained in Materials and Methods. At least 55 cells were analysed. * p<0,05 (Kruskal-Wallis test). (C) NIH/3T3 cell were transfected with plasmids coding for GFP-NFAT and either myc-MBP or myc-MBP-M9M. NFAT nuclear translocation was stimulated with inonomycin. Cells were stained for MBP or MBP-M9M (anti-myc, magenta) and nucleus (DAPI, blue) and NFAT was visualized by GFP fluorescence (green). Scale bar: 10 μm. (D) Nuclear NFAT in MBP/MBP-M9M expressing cells was quantified measuring the mean GFP fluorescence in the nuclear compartment as explained previously. The mean nuclear fluorescence was normalised against the mean total GFP fluorescence of the cell. At least 45 transfected cells were quantified. *** p<0.0001 (ANOVA). (TIF) [file pone.0162033.s005.tif]
